# Complete Low‐Temperature Transformation and Dissolution of the Three Main Components in Corn Straw

**DOI:** 10.1002/open.202200247

**Published:** 2023-02-01

**Authors:** Diyan Qin, Yancheng Liu, Ruofeng Yang, Jianmei Li, Changwei Hu

**Affiliations:** ^1^ Key Laboratory of Green Chemistry and Technology Ministry of Education College of Chemistry Sichuan University Chengdu Sichuan 610064 P. R. China

**Keywords:** aluminum chloride, cellulose, hemicellulose, lignin, solvothermal treatment

## Abstract

The conversion of biomass faces the challenge of mass and heat transfer, as well as the exertion of heterogeneous catalyst, because raw biomass exists usually in solid state. In this work, the simultaneous transformation and dissolution of the three main components (hemicellulose, cellulose, lignin) in corn straw were achieved in ethanol/ valerolactone (GVL)/H_2_O (10 : 10 : 40, *v*/*v*/*v*) co‐solvent system. With the assistance of AlCl_3_ ⋅ 6H_2_O, the conversion of hemicellulose, lignin and cellulose was >96 % at 170 °C. The conversion of solid biomass into fluid, overcoming the mass transfer restrictions between solid biomass and solid catalysts, provides new raw materials to further upgrading. H_2_O could penetrate inside the crystalline cellulose to swell even dissolve it, while ethanol and GVL acted as media to dissolve especially the G unit in lignin. The H^+^ derived from AlCl_3_ ⋅ 6H_2_O hydrolysis could break the linkages of lignin‐hemicellulose and glycosidic bond in saccharides, and aluminum chloride promoted the next degradation of polysaccharides to small molecules. Consequently, as high as 33.2 % yield of levulinic acid and 42.2 % yield of furfural were obtained. The cleavage of β‐O‐4 and C_β_−C_γ_ bonds in lignin produced large amounts of lignin‐derived dimers and trimers. The total yield of monomeric phenols is up to 8 %.

## Graphical abstract

**The complete conversion technology** of biomass developed in this paper transforms all the three components of solid biomass into fluids, not only obtaining the desired yield of small molecules, but also providing a favorable raw material for the subsequent upgrading, promoting heat and mass transfer, and facile separation of catalyst.

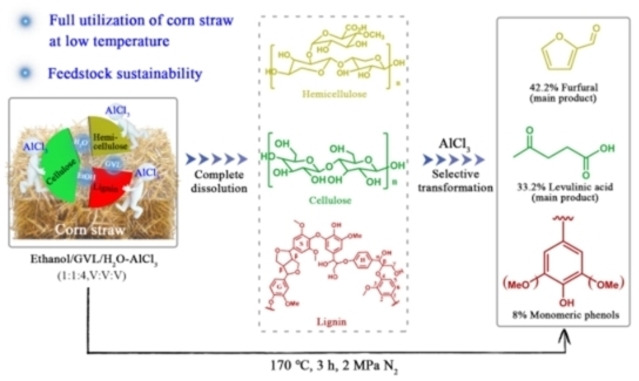

## Introduction

As a widely available and renewable resource, lignocellulosic biomass waste is a potential alternative of fossil resource for the production of fuels and chemicals without affecting the food supply.[^1^, ^2^, ^3^] It is reported that about 900 million tons of agricultural and forestry wastes are produced in China per year. This includes corn straw, which is usually burned and thus causes environmental pollution and damages to the ecological balance of the soil.[^4^, ^5^, ^6^] The effective utilization of such raw materials has aroused great interests of researchers. Biorefineries utilize various components of biomass and its intermediates to maximize the value derived from the biomass feedstock, and carbohydrates (cellulose and hemicellulose) and lignin have been widely used in bioenergy and chemicals, such as, industries related to food production, pulp and paper industries, liquid and gas biofuels for road transport, aviation and shipping, all of which are remarkable examples of promising bio‐refining.[^7^, ^8^, ^9^] Lignocellulose is mainly composed of lignin, cellulose and hemicellulose components with distinct structure and properties.[^10^, ^11^, ^12^] It is generally believed that hemicellulose is a heteropolymer composed of several different types of five‐carbon sugars and six‐carbon sugars, including mainly d‐xylose, d‐glucose, l‐arabinose, and d‐mannose, mainly d‐xylose.[^13^, ^14^] The hemicellulose discussed in the present manuscript referred a composite glycan determined by Van Soest titration^[15]^ to remove other reducing sugars except for starch, cellulose, and pectin. Among these three components, there is a large amount of hydrogen bonds and C−O‐C ether bonds.[^16^, ^17^, ^18^] The complex structure of lignocellulose and the large number of junctions and the presence of impurities such as inorganic salts make them resistant to dissolution and degradation.[^19^, ^20^, ^21^] The solid state with complex components of biomass inhibited its interaction with solid catalyst, and the mass transfer, heat transfer and momentum transfer in the reaction system are difficult, which makes the industrialization extremely difficult.[^22^, ^23^] It is highly desired to break the strong interaction between cellulose, hemicellulose and lignin, and transform the solid raw biomass into fluid, facilitating the further processes with high efficiency of mass and heat transfer, as well as the exertion of heterogeneous catalyst, promoting the industrialization of biomass utilization.

Considerable efforts have been endeavored to explore solvothermal system for the fractionation of lignocellulose. For example, supercritical carbon dioxide has been shown to be very effective in the fractionation and hydrolysis of lignocellulosic fractions. Under high pressure, CO_2_ easily permeates into the pores of refractory LCM, causing structural changes and enhancing the enzymatic hydrolysis accessibility of glucan and xylan.^[24]^ Water as a solvent can selectively utilize hemicellulose, recovering 53 % of xylose at 215 °C, while cellulose and lignin remain not utilized in the solid in solids.^[25]^ Besides, organic solvent is proved to play an important role. For instance, Philipp et al. developed an “organocatalytic” process for the separation of the three main components of lignocellulose in a H_2_O‐2‐methyltetrahydrofuran‐oxalic acid two‐phase system.^[26]^ This method allowed the depolymerization of hemicelluloses to form an aqueous stream of the corresponding carbohydrates. The dissolved lignin was almost extracted in the organic phase, while the cellulose was remained as solid residue. Dumesic et al. found that γ‐valerolactone (GVL) significantly accelerated the reaction rate and product selectivity in lignocellulose transformation when compared to water. GVL also promoted the hydrolysis of cellobiose to glucose.^[27]^ He et al. developed a ethanol/H_2_O (1/1, *v*/*v*) co‐solvent system with 0.05 m oxalic acid as the catalyst, achieving the simultaneous fractionation of hemicellulose and lignin components in corn straw with 88.0 wt % and 89.2 wt % conversion, respectively; while cellulose was not obviously degraded.^[28]^ On the basis of these previous studies, it was inferred that organic solvent might act as a nucleophile to promote the breakage of linkages between hemicellulose (such as ether and ester bonds) and lignin, and could also facilitate the dissolution of lignin fragments.

Inorganic salts (such as NaCl, ZnCl_2_, Na_2_CO_3_) are generally considered to promote lignocellulose dissolution and conversion, where the penetration of ions might disrupt the intermolecular and intramolecular hydrogen bond network. Liu et al. found that Na_2_CO_3_ destroyed almost all of the ether, ester, and hydrogen bonds between lignin and cellulose at 140 °C, resulting in a 94.6 % of lignin conversion.^[29]^ Jiang et al. indicated that NaCl greatly promoted the dissolution and degradation of cellulose into oligomers under relatively mild conditions. It was pointed out that Cl^−^ could interact strongly with the terminal hydroxyl group of glucose unit in a 1 : 1 ratio, resulting in the breakage of the intermolecular and intramolecular hydrogen bonds.^[30]^ The employment of ferric chloride as the catalyst enhanced the yield of total reduced sugar to 31.5 %.^[31]^

The co‐operative roles of solvent with salt have been proved to be a promising approach to overcome the complex structure and abundant linkages in lignocellulose transformation. However, the current strategies still confront great challenges. For instance, the distinct composition and properties of the three components in lignocellulose generally lead to incomplete dissolution of all the three components. In addition to the severe treatment conditions, complicated processing steps are usually required. In this study, we take advantages of both solvothermal system and salt, achieving the simultaneously complete transformation and dissolution of all the three main components in corn straw in GVL/ethanol/H_2_O (10 : 10 : 40, *v*/*v*/*v*) co‐solvent system with the assistance of AlCl_3_ ⋅ 6H_2_O. The complete conversion and treatment technology of biomass developed in this paper turns all the three components of solid biomass into fluids, which not only obtains small molecules with objective yield, but also provides raw material flow for subsequent processing and treatments, facilitating heat and mass transfer as well as the contact of the reactant with heterogeneous catalyst. It was indicated that corn straw conversion‐dissolution could be controlled by changing the ratio of solvent, salt concentration, reaction temperature and time. The roles of solvents and AlCl_3_ ⋅ 6H_2_O were comprehensively studied.

## Results and Discussion

### The dissolution and conversion of corn straw

Considering the sustainability and abundance of water, we first investigated the dissolution of corn straw in water (Table 1). Based on our previous results, we speculated that water may act as a nucleophile to promote the bonds between hemicellulose and lignin (such as ether bonds and ester bonds), as well as the breakage of some intermolecular and intramolecular hydrogen bonds in cellulose.^[32]^ Since lignin is insoluble in water, and the degradation of cellulose requires higher temperatures,[^33^, ^34^] the conversion rate of hemicellulose after water treatment is 42.4 %, while the conversion rates of cellulose and lignin are very low, 13.4 % and 2.0 %, respectively. Ethanol can increase the conversion rate of lignin and hemicellulose to 22.4 % and 54.2 %, whereas the increase of cellulose conversion rate is not obvious. GVL significantly improved the conversion rate of hemicellulose and lignin, and it also increased that of cellulose to 31.7 %. Nevertheless, the high cost and high boiling point of GVL made it difficult to be recycled, which was unfavorable for the separation of obtained liquid products for the next upgrading. Combined with our previous results^[35]^, we speculate that water may also attack the reaction center of lignin, “filling” −H and −OH at the cleavage site. The GVL and ethanol may contribute to the dissolution of the lignin fragments, which may serve as blocking reagents to protect against the repolymerization of the lignin units. So we adopted the GVL/ethanol/H_2_O cosolvent system.

**Table 1:** Dissolution of corn straw in different solvents.^[a]^

Solvent [mL]			Conversion [%]		
GVL	Ethanol	H_2_O	Lignin	Hemicellulose	Cellulose
0	0	60	2.0 (21.6)	42.4 (82.5)	13.4 (26.1)
0	60	0	22.4 (83.7)	54.2 (86.5)	14.4 (28.9)
60	0	0	47.1 (86.2)	68.8 (95.4)	31.7 (37.6)
10	10	40	26.1	62.7	16.0

[a] Conditions: 0.6 g corn straw (100 g L^−1^), 2.0 MPa N_2_, 130 °C, 3 h. The value in parentheses is obtained by adding aluminum chloride in the solvent (0.05 m).

We treated biomass by using solvent with adding aluminum chloride, and the results are shown in Table 1 and Table 2. We first investigated the dissolution of corn straw in water medium in the presence of AlCl_3_ ⋅ 6H_2_O. The results showed that 82.5 % of hemicellulose in corn straw was dissolved at 130 °C, while the dissolution of lignin and cellulose components was quite limited. For comparison, the conversion of corn straw in single ethanol or GVL with aluminum chloride was also studied. It was found that single ethanol solvent facilitated the dissolution of both hemicellulose and lignin components, the conversion of which was 86.5 % and 83.7 %, respectively. However, the conversion of cellulose was quite low. In the case of single GVL solvent, 95.4 % hemicellulose and 86.2 % lignin were transformed. Overall, the conversion rates of all three components were significantly improved after the addition of aluminum chloride in the solvents.

**Table 2:** Dissolution of corn straw in various solvent systems.^[a]^

Solvent [mL]			Conversion [%]		
GVL	Ethanol	H_2_O	Lignin	Hemicellulose	Cellulose
0	0	60	21.6	82.5	26.1
0	5	55	43.1	88.8	27.5
5	5	50	65.4	90.6	34.1
10	10	40	88.3	92.2	34.4
20	20	20	83.2	88.5	29.1
0	60	0	83.7	86.5	28.9
5	50	5	85.1	90.6	24.8
60	0	0	86.2	95.4	37.6
50	5	5	88.6	94.9	34.6

[a] Conditions: 0.6 g corn straw (100 g L^−1^), 0.7243 g AlCl_3_ ⋅ 6H_2_O (0.05 m), 2.0 MPa N_2_, 130 °C, 3 h.

**Figure 1:**
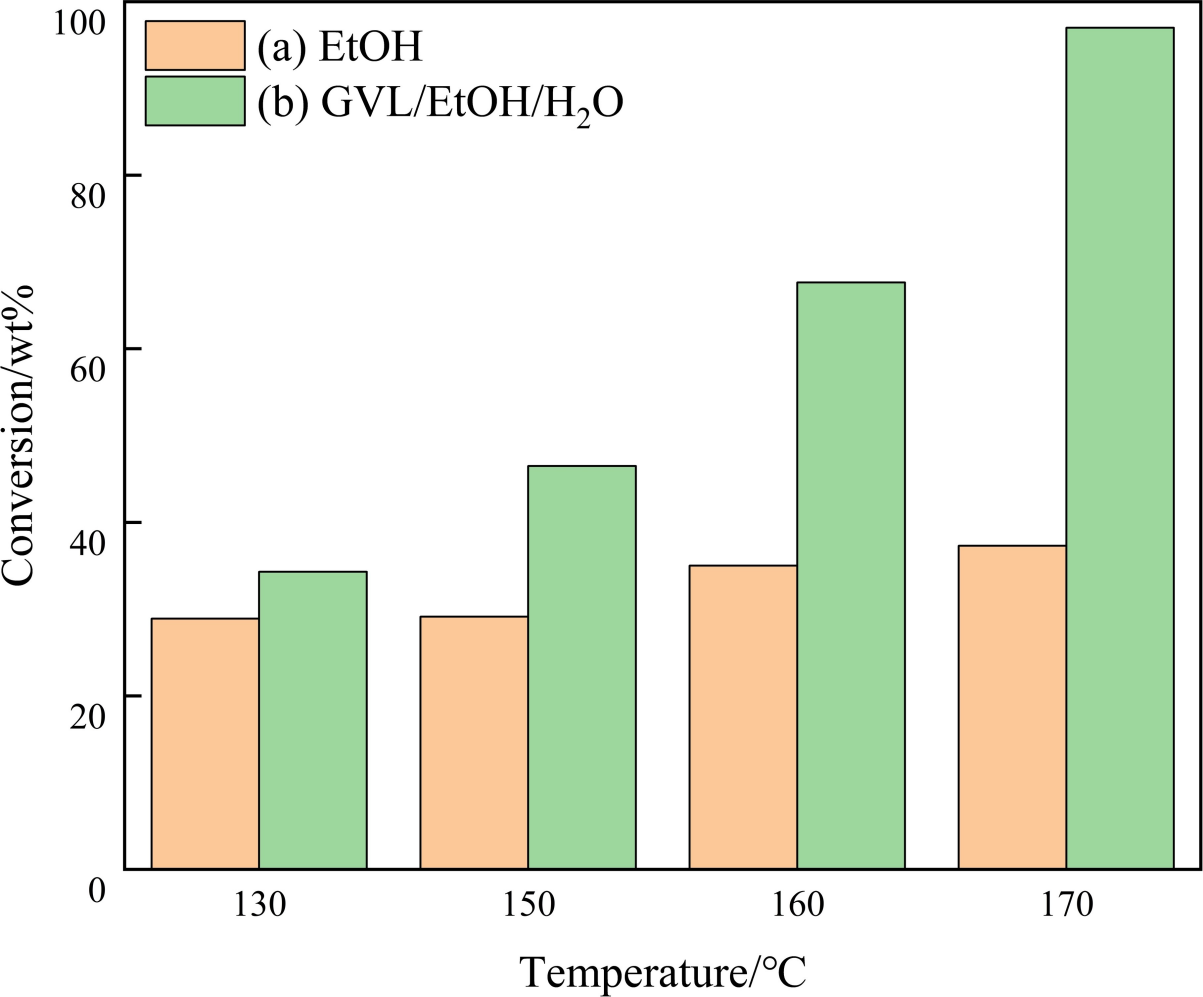
Conversion of cellulose with temperature in different solvents. (a) 60 mL EtOH, (b) ethanol/GVL/H_2_O (10 : 10 : 40, *v*/*v*/*v*, 60 mL total volume). Conditions: 0.6 g corn straw (100 g L^−1^), 0.05 m AlCl_3_ ⋅ 6H_2_O, 2.0 MPa nitrogen, 3 h.

To further improve the dissolution of lignin component, ethanol‐water co‐solvent was then employed. It was found that a small amount of ethanol introduction (ethanol:H_2_O=5 : 55, *v*/*v*) improved lignin conversion from 21.6 % to 43.1 %. When the GVL‐ethanol‐H_2_O co‐solvent was employed, the conversion of lignin further increased to 65.3 % at a ratio of 5 : 5 : 50 (GVL/ethanol/H_2_O, *v*/*v*/*v*), while the conversions of hemicellulose and cellulose were slightly improved to 90.6 % and 34.1 %, respectively. Increasing the contents of GVL and ethanol to 10 : 10 : 40 (GVL/ethanol/H_2_O, *v*/*v*/*v*) led to the obvious increase of lignin conversion to 88.3 %, as well as the slight increase of hemicellulose conversion to 92.2 %. These results indicated that aluminum chloride and GVL/ethanol/H_2_O co‐solvent might exhibit a synergetic effect for the dissolution of hemicellulose and lignin in corn straw. Hemicellulose and lignin and non‐crystalline cellulose are amorphous structures, which are relatively easy to depolymerize, the difficulty is the transformation of crystalline cellulose. The current conditions seem that ethanol also works very well. As shown in Figure 1, when the temperature was increased to 170 °C, the cellulose conversion rate was only increased to 37.3 % in EtOH, and the ethanol/GVL/H_2_O cosolvent system could convert almost all of the cellulose. This is because the crystalline cellulose has high crystallinity, dense hydrogen bonds, so it is more difficult to be transformed. However, the water molecules can penetrate into the fiber crystallization zone to produce infinite swelling and dissolve the cellulose. In addition, GVL has been proved to be a good solvent for the dissolution and transformation of cellulose. Considering the economy and feasibility, we chose the solvent with ethanol, GVL, and water at volume ratio of 10 : 10 : 40 for further study.

**Figure 2:**
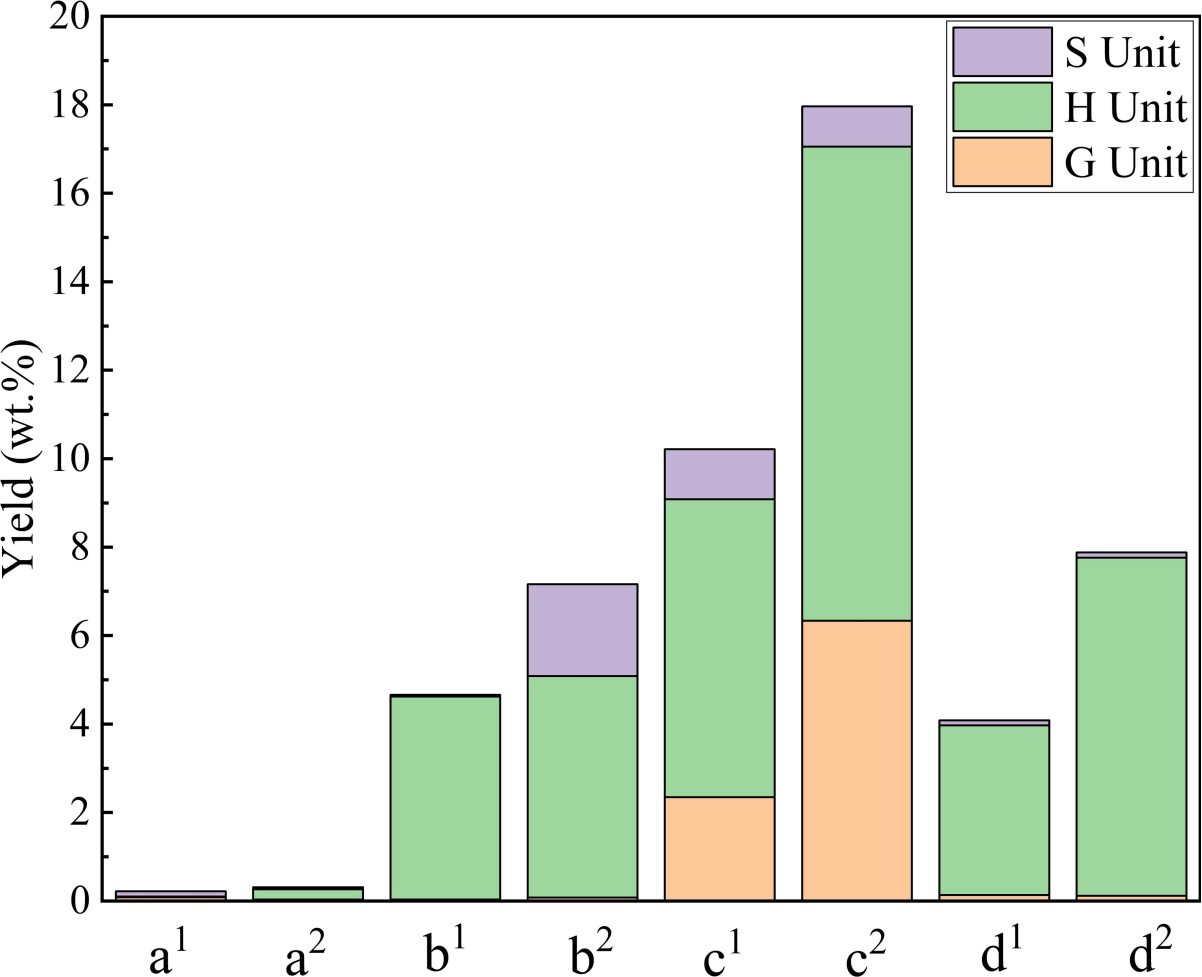
The yield of aromatic monomers in different solvents. (a) 60 mL H_2_O, (b) 60 mL EtOH, (c) 60 mL GVL, (d) ethanol/GVL/H_2_O (10 : 10 : 40, *v*/*v*/*v*, 60 mL total volume). Conditions: 0.6 g corn straw (100 g L^−1^), 130 °C, 0.05 m AlCl_3_ ⋅ 6H_2_O, 2.0 MPa nitrogen, 3 h. ^1^ The yield obtained with only the solvent. ^2^ The yield obtained by treatment with a solvent containing aluminum chloride.

The depolymerization of lignin led to the formation of phenolic monomers. The aromatic monomer products were quantitatively analyzed by gas chromatography (GC) to calculate the yield. As can be seen from Figure 2, the yield of monophenol in water was very low (0.22 %), and the yield did not increase significantly after aluminum chloride was added (0.31 %). When the solvent was changed to ethanol, the yield of phenolic monomer of H unit was up to 4.6 %, but the yield of G unit and S unit was still very low. When aluminum chloride was added to ethanol, the yield of S unit increased to 2.1 %. The total yield of monophenols treated with GVL was 10.2 %, which reached 18.0 % when aluminum chloride was added to GVL. Using a mixture of the three solvents (adding 10 mL ethanol and 10 mL GVL to 40 mL water) achieved a 4.1 % monophenol yield, which was higher than in pure water. After addition of aluminum chloride, the monophenol yield increased to 7.9 %, which was higher than in pure water and in ethanol, but lower than in GVL. These indicate that GVL, ethanol, co‐act with aluminum chloride promote monophenol production, but GVL does the best.

**Figure 3:**
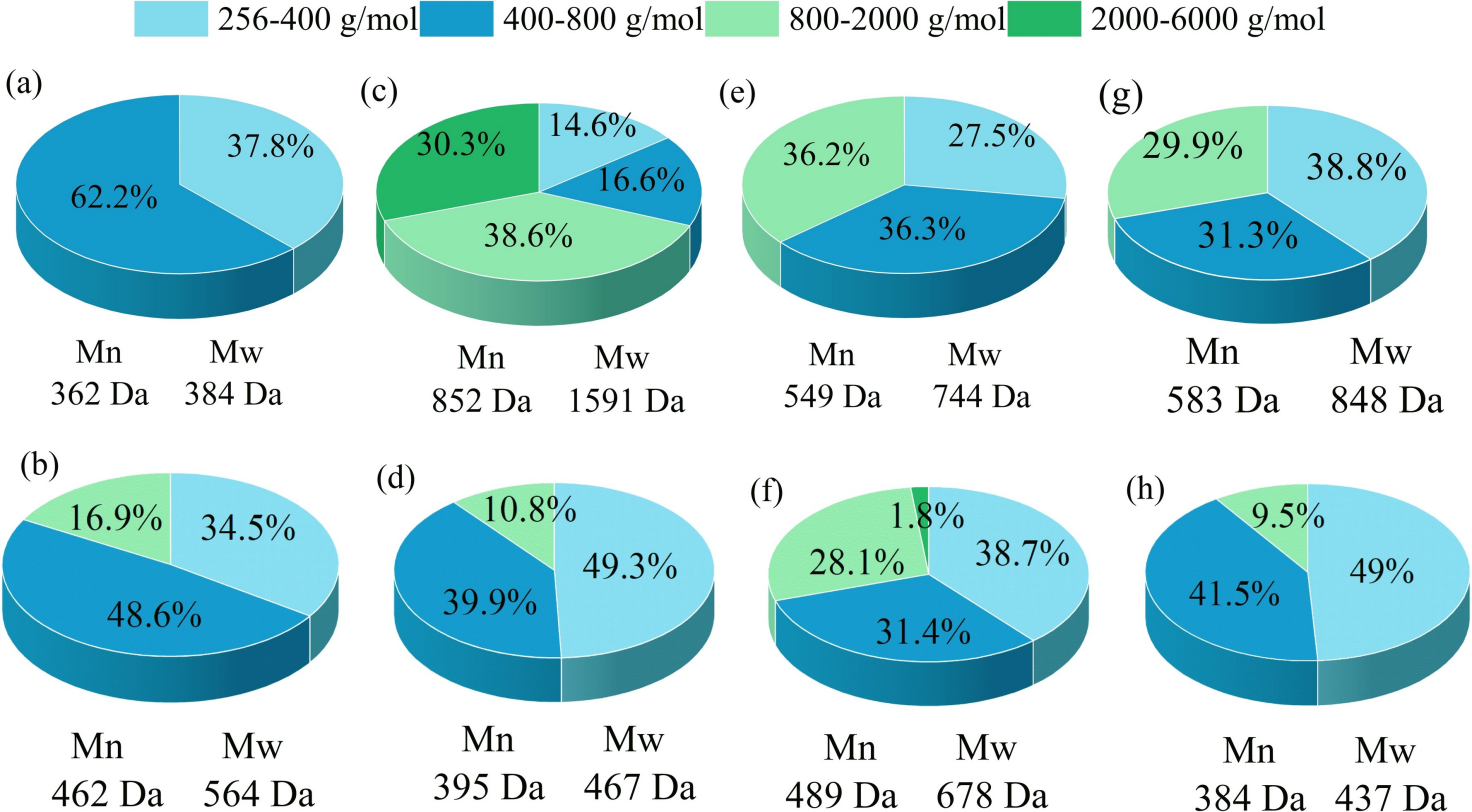
The molecular‐weight distribution of liquid products obtained from different solvent treatments. (a‐b) 60 mL H_2_O, (c–d) 60 mL EtOH, (e‐f) 60 mL GVL, (g–h) ethanol/GVL/H_2_O (10 : 10 : 40, *v*/*v*/*v*, 60 mL total volume). Conditions: 0.6 g corn straw (100 g L^−1^), 130 °C, 0.05 m AlCl_3_ ⋅ 6H_2_O (only in (b) (d) (f) (h) ), 2.0 MPa N_2_, 3 h. (a) (c) (e) (g) were treated with only solvent, and (b) (d) (f) (h) were added to the solvent with aluminum chloride.

The liquid treated with different solvents was lyophilized to remove water and then GPC analysis was performed to observe the molecular weight distribution of the oligomers. The result is shown in Figure 3. Due to the low lignin conversion rate of 2.0 % in pure water, the main composition of oligomers at this time is polysaccharide. The weight‐average (M_w_) and number‐average (M_n_) of oligomers were 384 and 362 Da, respectively, indicating that most oligosaccharides existed in the form of dimeric sugars at this time. Ethanol and GVL can dissolve a large amount of lignin, but the yield of monophenols is very low, so most lignin still exists in the form of oligomers in the liquid. 68.9 % of the oligomers dissolved in ethanol were distributed in the range of 800–6000 g mol^−1^, and 72.5 % of the oligomers dissolved in GVL were distributed in the range of 400–2000 g mol^−1^. Using a mixture of the three solvents (adding 10 mL ethanol and 10 mL GVL to 40 mL water) can increase the distribution of 256–400 g mol^−1^ up to 38.8 %. It is worth noting that very few sugar‐derived small molecules are obtained after treating the biomass with the solvent alone, so some of the oligomers are oligosaccharides. The molecular weight of the oligomers greatly decreased after the addition of aluminum chloride. In the mixed solvent, the molecular weight decreased the most, and the number‐average molecular weight was as low as 384 Da, indicating that the oligomers were mainly dimers and trimers.^[29]^

According to the above results, it is inferred that ethanol can promote the dissolution of the H unit. GVL can greatly improve the monomer yield of G, S and H units. Lignin fragments were more likely to be broken into small molecules in GVL than in ethanol. Aluminum chloride can promote the fracture of lignin crosslinking units, making lignin fragments smaller and increasing the yield of monophenols.

### Optimization of conversion and dissolution conditions

**Figure 4:**
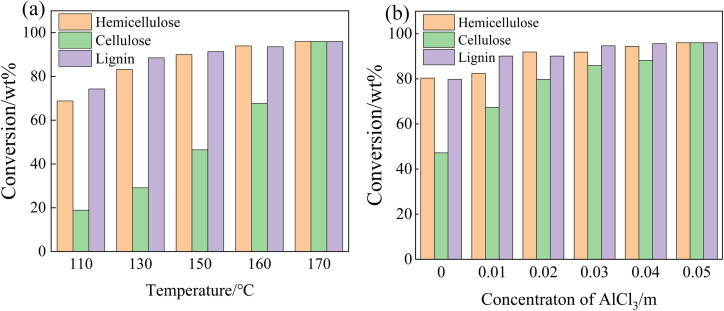
The dissolution of corn straw at different temperatures (a) and AlCl_3_ ⋅ 6H_2_O concentrations (b) in GVL/ethanol/H_2_O system (10 : 10 : 40, *v*/*v*/*v*, 60 mL). Reaction conditions: (a) 0.6 g corn straw (100 g L^−1^), 0.05 m AlCl_3_ ⋅ 6H_2_O, 2.0 MPa N_2_, 3 h; (b) 0.6 g corn straw (100 g L^−1^), 170 °C, 2.0 MPa N_2_, 3 h.

It is generally known that cellulose has high crystallinity, and high temperature (180‐220 °C) is usually required to dissolve cellulose.[^36^, ^37^] The effect of temperature on the dissolution of cellulose, hemicellulose, and lignin in GVL/ethanol/H_2_O co‐solvent system was tested. As shown in Figure 4a, the conversions of cellulose, hemicellulose, and lignin at 110 °C were 18.9 %, 68.8 %, and 74.3 %, respectively. With increasing temperature, the conversion of cellulose sharply increased, while the conversions of lignin and hemicellulose just increased slightly. At 170 °C, 97 % of corn straw was converted and dissolved with only 3 % of residue (Figure 4b).

The AlCl_3_ ⋅ 6H_2_O concentration also affected the conversion of corn straw, especially for the cellulose component. With the introduction of a small amount of AlCl_3_ ⋅ 6H_2_O (0.01 m), the conversions of cellulose, lignin and hemicellulose were 67.3 %, 82.4 % and 90.1 %, respectively. Further increasing the AlCl_3_ ⋅ 6H_2_O concentration induced the gradual increase of cellulose conversion, while the conversions of hemicellulose and lignin slightly increased. In the presence of 0.05 m AlCl_3_ ⋅ 6H_2_O, the three components in corn straw could be almost completely dissolved. Owing to the hydrolysis of AlCl_3_ ⋅ 6H_2_O in aqueous solution, it was speculated that Cl^−^ in the reaction system could destroy the intermolecular and intramolecular hydrogen bonds in cellulose, thus greatly improving the solubility of cellulose.^[38]^ The resultant H^+^ could attack the O atom of the 1,4‐glycosidic bond, and then the protonated glycosidic bond was broken to form carbocation. H^+^ subsequently quickly transferred to carbocation, leading to the depolymerization of saccharides. As a typical Lewis acid, Al^3+^ in AlCl_3_ ⋅ 6H_2_O might promote the isomerization of aldose derived from corn straw to ketose, leading to the formation of a minority of small molecules.

### Characterization of the solid residue

**Figure 5:**
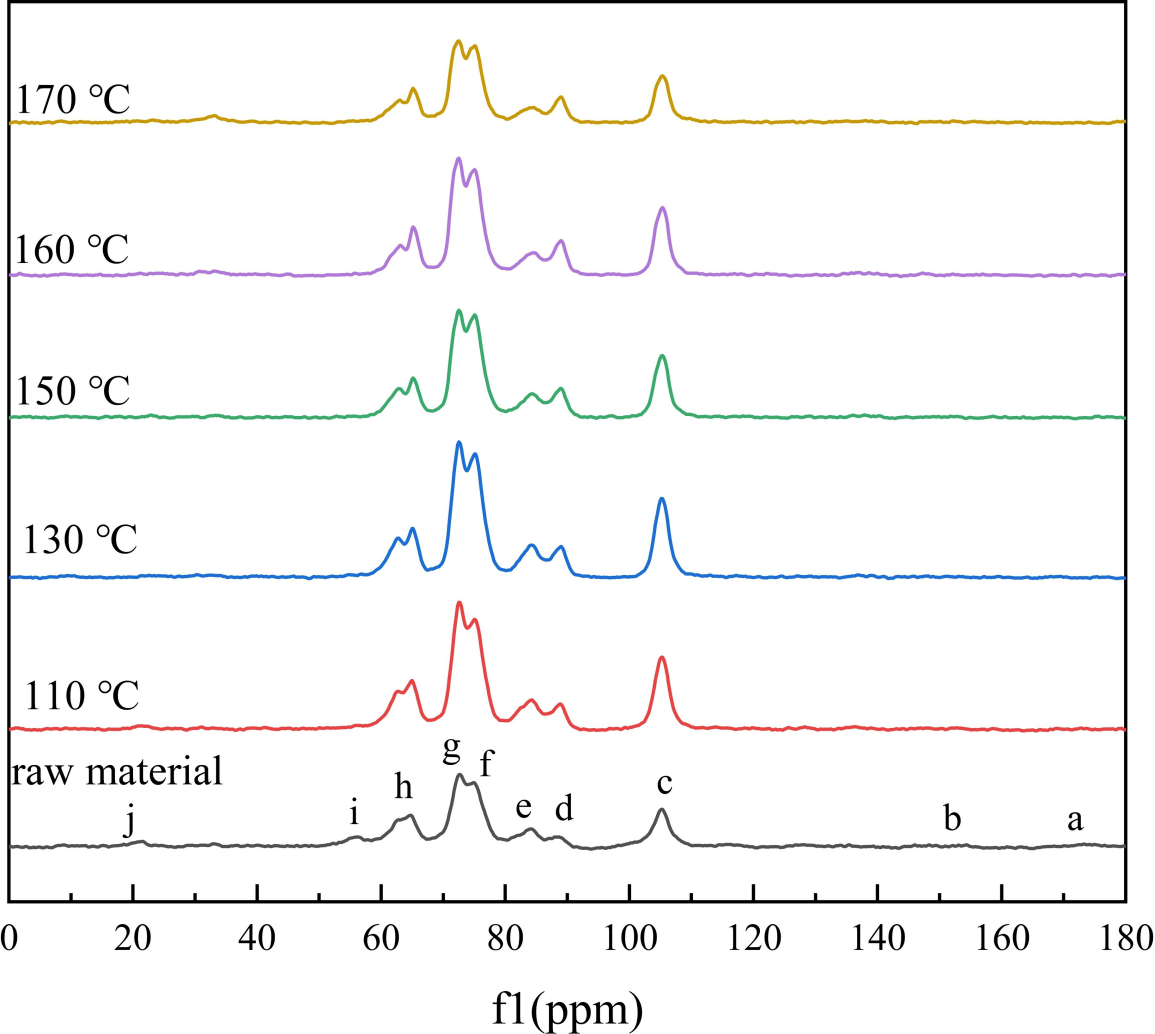
^13^C CP/MAS NMR spectra of corn straw and solid reaction residue treated in water GVL/ethanol/H_2_O (10 : 10 : 40, *v*/*v*/*v*) system at different temperatures. Reaction conditions: 0.6 g corn straw (100 g L^−1^), 0.05 m AlCl_3_ ⋅ 6H_2_O, 2.0 MPa N_2_, 3 h.

Solid‐state ^13^C CP/MAS NMR spectroscopy was used to analyze the raw material and solid reaction residues treated in GVL/ethanol/H_2_O (10 : 10 : 40, *v*/*v*/*v*) system at different temperatures (Figure 5). As for raw corn straw, obvious signals corresponding to cellulose, hemicellulose and lignin components were observed. The strong peaks with chemical shift at 73 ppm (g) and 75 ppm (f) were overlapping signals of C‐2, C‐3, and C‐5 of all polysaccharides. The chemical shift at 83 ppm (e) was assigned to C‐4 of amorphous cellulose and hemicellulose.^[39]^ The signals at 65 ppm (h) and 89 ppm (d) were corresponded to C‐6 and C‐4 of crystalline cellulose, respectively. The peak at 105 ppm (c) was specified as cellulose coronal carbon (C‐1). The signals at 168–178 ppm (a) were associated to the carbonyl groups of hemicelluloses and lignin. The peak at 20 ppm (j) was assigned to the acetyl group of hemicellulose.[^40^, ^41^, ^42^]

The peaks at 56 ppm (i) and 130–155 ppm (b) were assigned to methoxyl and aromatic ring in lignin, respectively. When corn straw was treated in the GVL/ethanol/H_2_O system, the signal intensity of the peaks at 20 and 55 ppm was weakened. This result further confirmed the removal of amorphous lignin and hemicellulose after treatment. In addition, it was found that the peak at 83 ppm (C‐4 of amorphous carbohydrate) gradually weakened with treatment temperature increasing above 130 °C, while the strength of peak at 89 ppm (C‐4 of crystalline cellulose) became stronger. In contrast, the signals corresponding to crystalline cellulose (e. g., C‐1, C‐4, C‐6) slightly strengthened, due to the higher content in solid residue after the removal of amorphous lignin and carbohydrate.^[43]^

**Figure 6:**
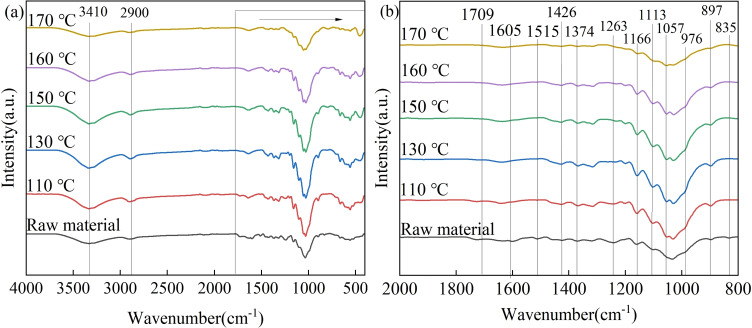
FTIR spectra of raw raw corn straw and solid residue treated in water GVL/ethanol/H_2_O (10 : 10 : 40, *v*/*v*/*v*) system at different temperatures. Reaction conditions: 0.6 g corn straw (100 g L^−1^), 0.05 m AlCl_3_ ⋅ 6H_2_O, 2.0 MPa N_2_, 3 h. (a) 4000–400 cm^−1^; (b) 2000–800 cm^−1^.

FTIR analysis was employed to further characterize the solid reaction residue (Figure 6). According to the reported literature, a wide absorption band at 3440–3200 cm^−1^ was attributed to phenolic and aliphatic OH groups, followed by bands for the C−H stretching in methyl and methylene groups (2900 cm^−1^). The peak at 1263 cm^−1^ was assigned to the C−O stretching of guaiacyl groups in lignin, while those at 1709, 1605 and 1515 cm^−1^ were the characteristic absorption peaks of lignin, corresponding to the aromatic skeleton vibration of lignin.[^44^, ^45^] The peak at 835 cm^−1^ corresponded to the aromatic C−H out of plane bending. The peaks at 1374, 1166, 1113 and 897 cm^−1^, corresponded to the C−H bending vibration, the C−O−C asymmetric stretching, asymmetric scaling and stretching vibrations of the pyran hexagon ring of glucose in cellulose component, respectively. Ferulic acids and *p*‐coumaric were considered responsible for the cross‐linkages through ester and ether bonds between lignin and hemicellulose, whose peak was found at 976 cm^−1^ (out of plane deformation of C=C in *p*‐coumaric and ferulic acids). Bands at 1605, 1515 and 835 cm^−1^ were considered to be the characteristic absorbance of structural benzene in lignin, while those at 1709, 1605, 1263 cm^−1^ were assigned to the characteristic stretching of hemicellulose.[^46^, ^47^] The appearances of peaks at 1426, 1374, 1263, 1057 and 897 cm^−1^ were assigned to the absorbance of cellulose. The characteristic peak of hemicellulose was at 1708 cm^−1^ and those for lignin at 1263 and 1515 cm^−1^, both of which decreased with the reaction temperature and disappeared at the reaction temperature of 170 °C, indicating that the hemicellulose and lignin in corn stover could be completely transformed at 170 °C, confirming the above deduction.

### Liquid products derived from hemicellulose and cellulose component

It was reported that AlCl_3_ aqueous solution could act as both Lewis acid and Brønsted acid due to its hydrolysis to generate hydroxyl‐Al^III^ species and H^+^. The Lewis acidity of hydroxyl‐Al^III^ species could promote the isomerization reaction such as xylose‐to‐xylulose, glucose‐to‐fructose, while the Brønsted acidity of H^+^ was favorable for the breakage of the glucosidic bond and the dehydration reaction, leading to the further transformation of dissolved species to various small molecules.^[48]^ After treatment in GVL/ethanol/H_2_O (10 : 10 : 40, *v*/*v*/*v*)‐AlCl_3_ ⋅ 6H_2_O system, the derivatives from hemicellulose and cellulose components were detected, including furfural, levulinic acid, xylose, formic acid, lactic acid, together with a quite small amount of arabinose, glyceraldehyde, acetic acid, eugenic acid, ethylene glycol (<3 % yield), and so on (Table 3). Xylose, furfural, and levulinic acid were the dominant products. As the temperature raise from 110 to 170 °C, the yield of xylose gradually decreased from 8.6 % to 3.2 %, possibly due to its further dehydration to furfural. In contrast, furfural yield sharply increased from 3.9 to 42.2 %. In addition, 17.9 % yield of levulinic acid was obtained at 110 °C, which was obviously raised to 33.1 % when the reaction temperature was increased to 170 °C. Raising the reaction temperature also increased the yield of formic acid, which was possibly produced accompanying with the formation of levulinic acid from 5‐hydroxymethylfurfural decomposition. As we know, 5‐hydroxymethylfurfural could be further converted to levulinic acid together with the formation of equivalent amount of formic acid.[^49^, ^50^] However, no 5‐hydroxymethylfurfural was detected in our work, possibly due to its further transformation to levulinic acid. Figure 4a shows that when the temperature increased from 110 to 170 °C, the cellulose conversion increased by 77.1 %, the hemicellulose conversion increased by 27.2 %, and the yield of hemicellulose derivatives mainly increased from 12.5 % to 45.4 %, while the yield of cellulose derivative increased from 22.2 % to 47.4 %. Therefore, xylose and furfural mainly come from the transformation of the dissolved species derived from hemicellulose, and lactic acid, formic acid and levulinic acid might be mainly derived from the solid cellulose via transformation and then dissolution. The remaining part dissolved in the system might be in the form of hexose‐derived oligomers, pentose‐derived oligomers, even humins. The overall mass balance of biomass reached 92.8 %, not including the gaseous products released. (The mass balance is calculated by dividing the sum of the liquid and solid masses after the reaction by the mass of the raw material). For difficulties for the quantification of oligomers in the fluid, the mass balance of each component (cellulose, hemicellulose, and lignin) is now not available.

**Table 3:** The effect of reaction temperature on the yield of small molecules derived from hemicellulose and cellulose components in corn straw.^[a]^

Temperature [°C]	Yield [wt %]				
	Xylose	Levulinic acid	Furfural	Formic acid	Lactic acid
110	8.6	17.9	3.9	1.5	2.8
130	4.1	18.3	38.2	2.6	2.2
150	3.5	23.5	39.7	4.8	2.6
160	3.2	28.0	41.2	5.7	2.8
170	3.2	33.1	42.2	10.0	4.3

[a] Conditions: 0.6 g corn straw (100 g L^−1^), GVL/ethanol/H_2_O (10 : 10 : 40, *v*/*v*/*v*, 60 mL total volume), 0.05 m AlCl_3_ ⋅ 6H_2_O, 2.0 MPa N_2_, 3 h.

We found that treating corn straw with each solvent alone produced sugar‐derived small molecule species with low yields, while which comes up after adding aluminum chloride. This shows that aluminum chloride plays a key role in promoting the next degradation of polysaccharides into small molecules. The influence of AlCl_3_ ⋅ 6H_2_O concentration on the yield was also investigated, and the results were shown in Table 4. It was demonstrated that the yield of levulinic acid and furfural first increased slightly with the increase of AlCl_3_ ⋅ 6H_2_O concentration, while it showed a sharp increase with AlCl_3_ ⋅ 6H_2_O concentration increased from 0.04 to 0.05 m. Compared to levulinic acid, a higher furfural yield was obtained at low AlCl_3_ ⋅ 6H_2_O concentration. In addition, the yield of xylose and formic acid slightly increased with increasing AlCl_3_ ⋅ 6H_2_O concentration, while lactic acid yield firstly increased and then decreased.

**Table 4:** The effect of AlCl_3_ ⋅ 6H_2_O concentration on the yield of small molecules derived from hemicellulose and cellulose components in corn straw.^[a]^

AlCl_3_ **⋅** 6H_2_O concentration [m]	Yield [wt %]				
	Xylose	Levulinic acid	Furfural	Formic acid	Lactic acid
0.01	0.7	4.4	10.5	6.4	7.4
0.02	1.2	10.1	25.3	9.3	7.6
0.03	3.0	11.5	27.1	9.0	8.0
0.04	3.0	13.3	29.1	10.6	9.1
0.05	3.2	33.1	42.2	10.0	4.3

[a] Conditions: 0.6 g corn straw (100 g L^−1^), GVL/ethanol/H_2_O (10 : 10 : 40, *v*/*v*/*v*, 60 mL total volume), 170 °C, 2.0 MPa N_2_, 3 h.

The gas products after treatment consisted of carbon dioxide, methane, hydrogen and carbon monoxide. Wherein, the main gas product is carbon dioxide (∼90 %), while the relative content of methane and hydrogen is low (<5 %). Carbon dioxide might originate from the decarboxylation of hemicellulose and cinnamic acid in lignin. The decomposition of formic acid also gave carbon dioxide and hydrogen, which was one of the possible reasons that formic acid yield was much lower than that of levulinic acid. Methane was probably produced by the breakdown of the methoxy group side chain in the lignin unit.

### Liquid products derived from lignin

**Figure 7:**
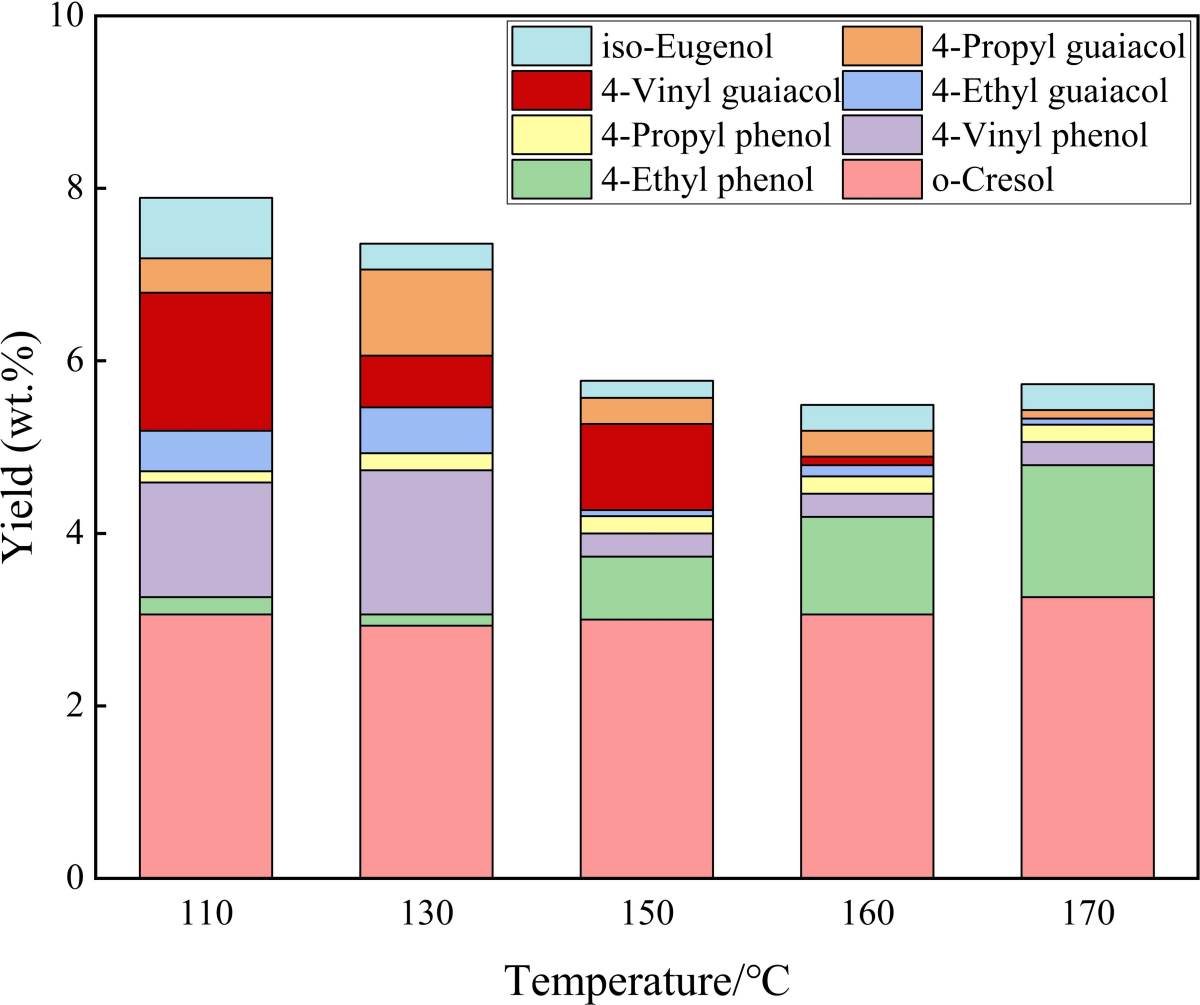
The yield of aromatic monomers. Conditions: 0.6 g corn straw (100 g L^−1^), GVL/ethanol/H_2_O (10 : 10 : 40, *v*/*v*/*v*, 60 mL total volume), 0.05 m AlCl_3_ ⋅ 6H_2_O, 2.0 MPa N_2_, 3 h.

The quantitative analysis of lignin‐derived products with small molecular weight was carried out by GC (Figure 7). The results showed that the identified phenolic monomers included o‐cresol, 4‐ethyl phenol, 4‐vinyl phenol, 4‐propyl phenol, 4‐ethyl guaiacol, 4‐propyl guaiacol, isoeugenol, but the amount of each monomer was quite low (<5 %). Although *p*‐coumaric acid or ferulic acid were reported to be obtained in the catalytic conversion of corn straw,^[51]^ they were not detected under the present conditions, possibly due to different raw material and/or different reaction conditions adopted. With the increase of reaction temperature, the yields of 4‐ethyl phenol and 4‐propyl phenol gradually increased. However, the yields of 4‐ethyl guaiacol, eugenol, 4‐vinyl phenol and 4‐vinyl guaiacol obviously decreased. The reduction of 4‐vinyl phenol and iso‐eugenol was possibly ascribed to the hydrogenation of double bonds in side‐chain, resulting in the formation of 4‐ethyl phenol and 4‐propyl guaiacol, respectively. The decrease of the yield of 4‐ethyl guaiacol might be attributed to dehydrogenation and decarbonylation or C_β_−C_γ_ bond breakage.[^52^, ^53^] The total yield of monophenol showed a slight decline with increasing reaction temperature, possibly due to the further esterification reaction with acetic acid derived from hemicellulose.

**Figure 8:**
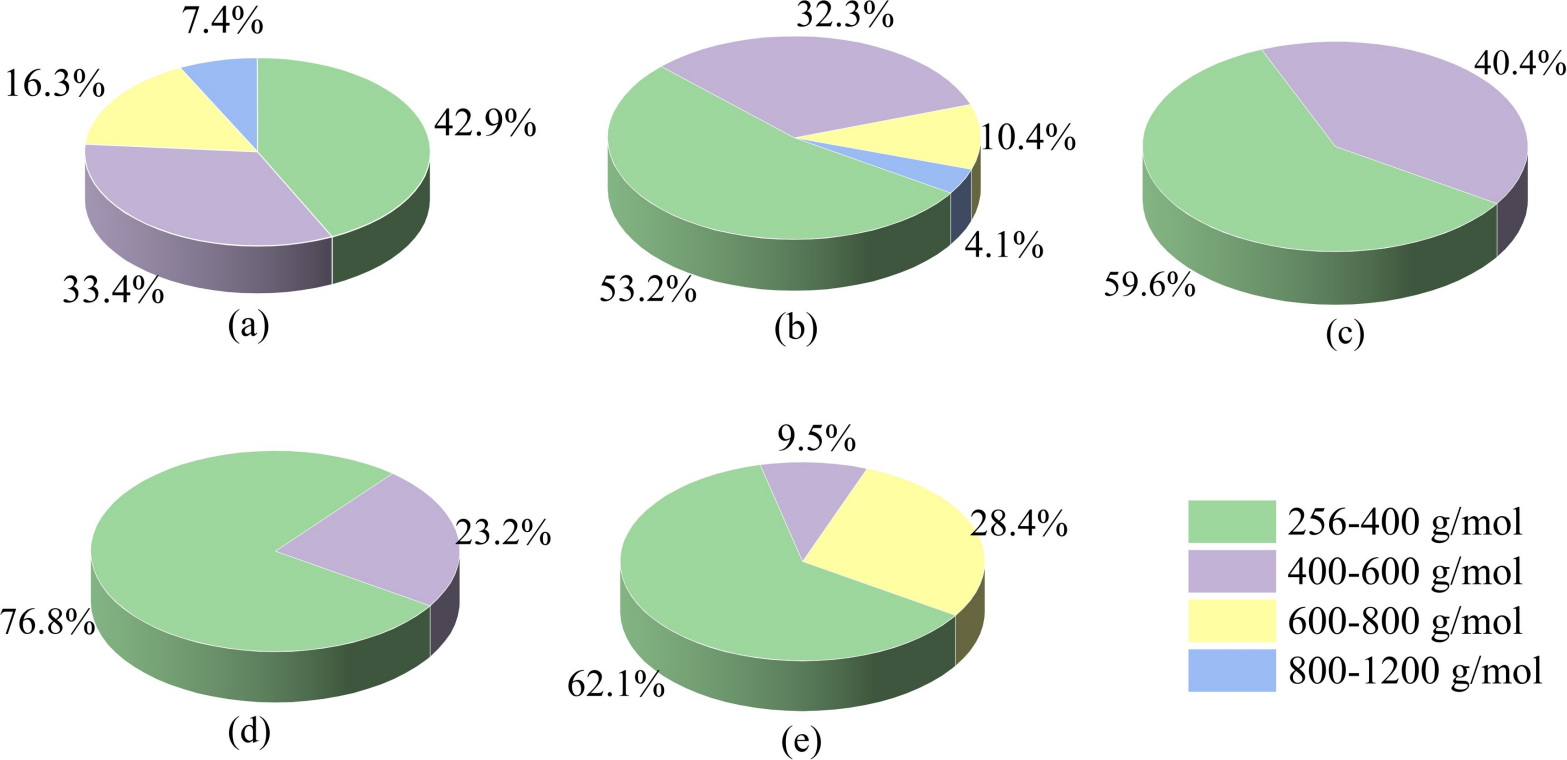
The molecular‐weight distribution of liquid products obtained by GPC analysis; (a)110 °C, (b) 130 °C, (c) 150 °C, (d) 160 °C and (e) 170 °C. Conditions: 0.6 g corn straw (100 g L^−1^), GVL/ethanol/H_2_O (10 : 10 : 40, *v*/*v*/*v*, 60 mL total volume), 0.05 m AlCl_3_ ⋅ 6H_2_O, 2.0 MPa N_2_, 3 h.

Given the almost complete removal of the lignin component in corn straw but the limited amount of phenolic monomers (∼10 %) obtained, the molecular weight distribution (mainly dissolved lignin fragments) in the liquid mixture was determined by GPC analysis after removal of water by lyophilization (Figure 8). It was found that the majority of oligomers (>70 %) treated at different temperatures had the molecular weight of 256–600 g mol^−1^, while the oligomers with molecular weight in a range of 600–1200 g mol^−1^ were less than 30 %. When the reaction temperature increased from 110 to 150 °C, the proportion of oligomers with high molecular weight (600‐1200 g mol^−1^) decreased gradually, while the fraction of low‐molecular‐weight oligomers (256–600 g mol^−1^) increased. At 150 °C and 160 °C, nearly all the molecular weight of the obtained oligomers was concentrated in the range of 256–600 g mol^−1^ (corresponding to dimer or trimers). The weight‐average (M_w_) and number‐average (M_n_) molecular weight of the oligomers also decreased slightly with the increase of reaction temperature to 160 °C (Table 5). When the reaction temperature was further raised to 170 °C, the proportion of oligomers with molecular weight less than 600 g mol^−1^ showed a slight decline, while some oligomers with molecular weight >600 g mol^−1^ was detected. The M_w_ and M_n_ also slightly increased. These results demonstrated that the dissolution of lignin in GVL/ethanol/H_2_O (10 : 10 : 40, *v*/*v*/*v*) system accompanied with the depolymerization to yield oligomers like dimer and trimmers. However, high temperature (170 °C) also caused the repolymerization of unstable oligomers.^[54]^

**Table 5:** Weight‐average (M_w_) and number‐average (M_n_) molecular weights and polydispersity (M_w_/M_n_) of the oligomers.^[a]^

	Temperature [**°C]**				
	110	130	150	160	170
M_n_/Da	421	384	347	331	358
M_w_/Da	479	437	369	344	383
Polydispersity	1.14	1.14	1.06	1.04	1.07

[a] Conditions: 0.6 g corn straw (100 g L^−1^), ethanol/GVL/H_2_O (10 : 10 : 40, *v*/*v*/*v*, 60 mL total volume), 0.05 m AlCl_3_ ⋅ 6H_2_O, 2.0 MPa N_2_, 3 h.

**Figure 9:**
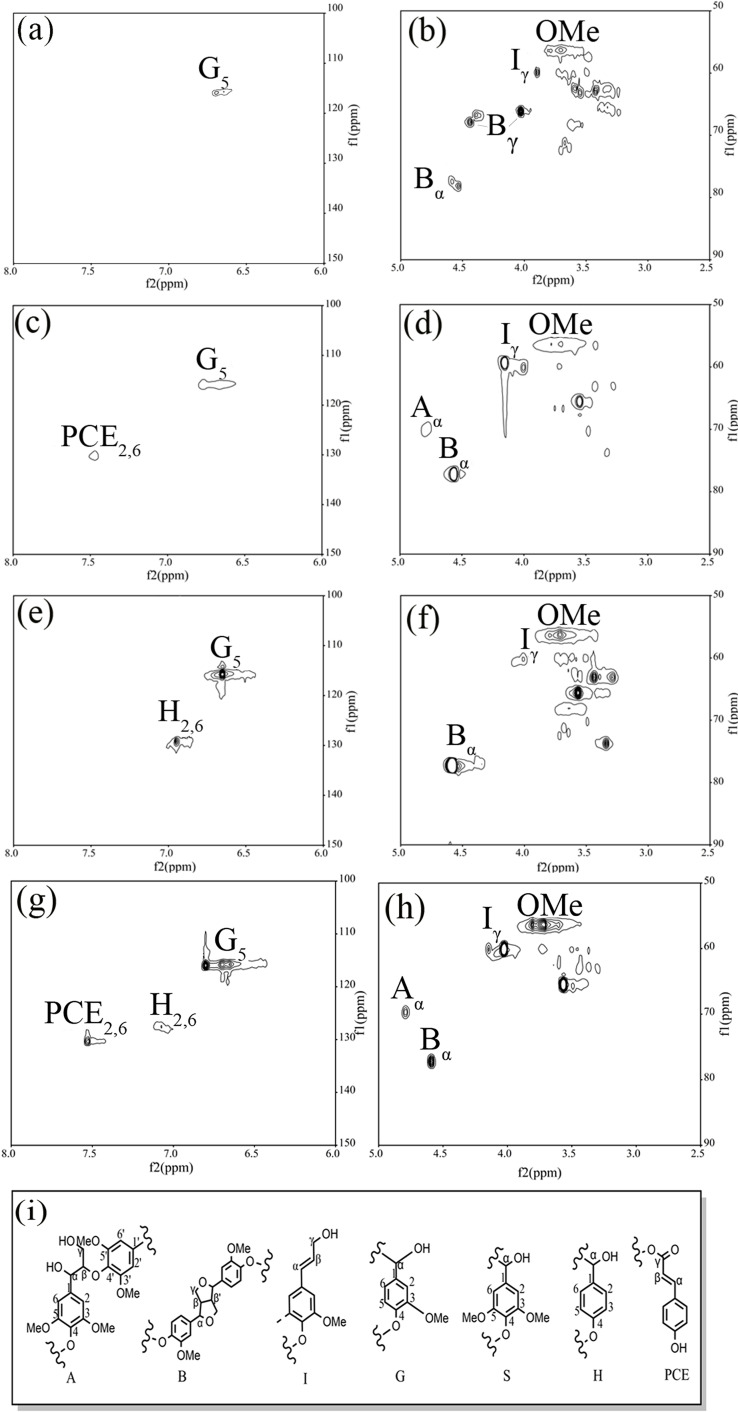
Aromatic regions and aliphatic side‐chain regions in the 2D HSQC NMR spectra of the liquid mixture obtained in organic phase after treatment in different solvent systems. (a–b) 60 mL H_2_O; (c–d) 60 mL GVL; (e–f) 10 mL ethanol, 10 mL GVL and 40 mL H_2_O; (g–h) 50 mL EtOH and 5 mL H_2_O and 5 mL GVL; (i) the relevant structure of lignin units. Conditions: 0.6 g corn straw (100 g L^−1^), GVL/ethanol/H_2_O (10 : 10 : 40, *v*/*v*/*v*, 60 mL total volume), 0.05 m AlCl_3_ ⋅ 6H_2_O, 170 °C, 2.0 MPa N_2_, 3 h.

2D HSQC NMR analysis was then employed to deeply reveal the structure of lignin‐derived oligomers obtained, as well as understanding the performance of solvent on the cleavage of the linkages among lignin units. With respect to the C−H signal of benzene ring in lignin unit, the main characteristic peak was G_5_ (Figure 9). The signal of C_2,6_−H_2,6_ assigned to the H unit was detected at 128.62/6.95 ppm (H_2,6_), but the signal of the syringyl unit (S) was not detected. The C_2,6_−H_2,6_ signal for coumaric acid was detected at 125.95/7.48 ppm (PCE_2,6_).^[55]^ This was consistent with the fact that herbs contained a certain amount of anticoumaric acid with less S component (compared to other kinds of biomass).[^56^, ^57^] As for the side chain of the lignin benzene ring, the strongest signal was observed for the −OCH_3_ on the benzene ring unit. In addition, the signals of β‐O‐4 structure were observed at 70.27/4.79 ppm (A_α_) and 60.18/3.66 ppm (A_γ_), while the obvious signal of β‐β structure was detected at 77.87/4.60 ppm (B).[^58^, ^59^, ^60^] β‐O‐4 linkage showed the highest signal strength, followed by β‐β linkage. It was speculated that the large number of G and H structural units in the dissolved lignin were primarily connected by β‐O‐4 manner. This was well consistent with the result in literature that very few β‐β and β‐5 connections to lignin side chains were found in maize stem biomass.^[35]^

The 2D HSQC NMR spectra (Figure 9 **)** of the liquid mixture treated in different solvents were analyzed. Compared to that treatment in ethanol/GVL/H_2_O cosolvent, it was found that the signals of coumaric acid (PCE_2,6_) and structure A (A_α_) disappeared when the solvent contained high amounts of water. In addition, the signals of H_2,6_ weakened or even disappeared, while the signals of B_γ_ appeared. When the solvent was GVL, the H_2,6_ signal also disappeared. However, in the case of ethanol as the main solvent, the signal of H_2,6_ could be observed. These results showed that water mainly destroyed β‐O‐4 and coumaric acid in lignin. GVL and water had a synergistic effect on the dissolution and transformation of H unit, whereas ethanol failed to dissolve the H structure.

The above characterizations show that, in addition to significant amounts of small molecular products obtained, the fluid obtained from the conversion and dissolution of solid biomass is mainly composed of dimers and trimers of monosaccharides and lignin units, which could be one favorable stream for further upgrading. In fact, the catalytic conversion of dimers and trimers have been widely investigated in previous studies to mimic biomass conversion,[^61^, ^62^, ^63^] while in the present work, dimers and trimmers are obtained directly from raw biomass, representing the actual structure of raw biomass. Thus another fractionation method of biomass conversion starting from the fluid obtained might be possible overcoming the bottleneck of engineering obstacles of mass and heat transfer as well as the use of solid heterogeneous catalysts in previously studied fractionation starting from solid actual biomass.

## Conclusions

With the assistance of AlCl_3_ ⋅ 6H_2_O, >96 % of hemicellulose, lignin and cellulose could be dissolved and transformed in ethanol/GVL/H_2_O co‐solvent system. Using waste biomass as a raw material, it provides a homogeneous raw material for the research of biomass and catalysts to realize the industrial transformation of biomass utilization. Ethanol and valerolactone played important roles in the dissolution and degradation of lignin into lignin fragments, while H_2_O molecules could swell the crystalline cellulose. AlCl_3_ ⋅ 6H_2_O hydrolyzed to generate H^+^ and hydroxyl‐Al^III^. H^+^ could break the linkages of lignin‐hemicellulose and the glycosidic bond in saccharides, and hydroxyl‐Al^III^ promoted the next degradation of saccharides to small molecules. Consequently, as high as 33.2 % yield of levulinic acid and 42.2 % yield of furfural were obtained. Under the synergetic actions of the solvent and AlCl_3_, the cleavage of β‐O‐4 and C_β_‐C_γ_ bonds in lignin led to the formation of 8 % monophenols and large amounts of lignin‐derived dimers and trimers.

## Experimental Section

### Materials

Corn straw was obtained from Fushun, Liaoning Province in China. Before use, it was ground into powder (40‐80 mesh), and dried in an oven at 80 °C. Ethanol (>99 %), γ‐valerolactone (GVL, 99 %), AlCl_3_ ⋅ 6H_2_O (>99 %), and other reagents for titration analysis were obtained from J&K Science Co., LTD. (Beijing, China). Tetrahydrofuran (THF) and methanol were purchased from Adamas‐Beta Chemical Reagents Factory. N_2_ (99.999 %) was provided by Chengdu Taiyu Natural Gas Company. All chemicals were used from commercial supply without further purification. Ultrapure water (18.25 Ω m) (AKexceed‐DC‐08, CTK) was used in all experiments.

### Solvothermal treatment

As for a typical reaction, 0.6 g corn straw powder, 60 mL solvent (10 mL ethanol, 10 mL GVL and 40 mL water), and 0.7243 g AlCl_3_ ⋅ 6H_2_O (0.05 m) were added in a 100 mL stainless steel autoclaves equipped with a mechanical whisk. Before reaction, the air in the reactor was replaced by N_2_, and the initial pressure of N_2_ was kept at 2.0 MPa. Subsequently, the reactor was heated to the designated temperature (110‐170 °C) with a stirring rate of 400 rpm for 3 h (Once the reactor was heated to the designated temperature, the reaction time was recorded). When the reaction was finished, the reactor was cooled to room temperature. The gas products obtained was collected for the subsequent analysis. After separating the solid residue from the liquid phase by filtration, the residual solid was washed with a small amount of distilled water until the cleaning liquid is neutral. The solid residue obtained was dried in an oven at 80 °C and weighed for subsequent analysis. The reaction solution was collected in a sample tube for further analysis.

The conversion of the reactants and the yield of products were defined by the following Equations (1)–4:

(1) $$\mathrm{Lignin}\;\mathrm{conversion}\;\left(\mathrm{wt}/\%\right)=\;\frac{\mathrm{Lignin}\mathrm{mass}\mathrm{in}\mathrm{reactant}-\mathrm{Lignin}\mathrm{mass}\mathrm{in}\mathrm{residue}}{\mathrm{Lignin}\mathrm{mass}\mathrm{in}\mathrm{reactant}}\times 100\;\%$$

(2) $$\mathrm{Cellulose}\;\mathrm{conversion}\;\left(\mathrm{wt}/\%\right)=\;\frac{\mathrm{Cellulose}\mathrm{mass}\mathrm{in}\mathrm{reactant}-\mathrm{Cellulose}\mathrm{mass}\mathrm{in}\mathrm{residue}}{\mathrm{Cellulose}\mathrm{mass}\mathrm{in}\mathrm{reactant}}\times 100\;\%$$

(3) $$\mathrm{Hemicellulose}\;\mathrm{conversion}\;\left(\mathrm{wt}/\%\right)=\;\frac{\mathrm{Hemicellulose}\mathrm{mass}\mathrm{in}\mathrm{reactant}-\mathrm{Hemicellulose}\mathrm{mass}\mathrm{in}\mathrm{residue}}{\mathrm{Hemicellulose}m\mathrm{ass}\mathrm{in}\mathrm{reactant}}\times 100\;\%$$

(4) $$\mathrm{Yield}\;\left(\mathrm{wt}/\%\right)=\;\frac{\mathrm{Weight}\mathrm{of}\mathrm{one}\mathrm{monomer}}{\mathrm{Weight}\mathrm{of}\mathrm{the}\mathrm{corresponding}\mathrm{precursor}\mathrm{in}\mathrm{corn}\mathrm{straw}}\times 100\;\%$$

The precursor of lactic acid, formic acid and levulinic acid is supposed to be cellulose, and that of xylose and furfural is hemicellulose, while that of the monophols is lignin.

#### Characterization

Van Soest titration method^[15]^ was used to analyze the composition of raw corn straw and solid residue after reaction. The ^13^C Cross‐Polarization Magic Angle Spinning Nuclear Magnetic Resonance (CPMAS NMR) analysis for the corn straw and solid residue was conducted on a BRUKER ADV ANCE III 500 MHz instrument.

The liquid products with short carbon chain derived from cellulose and hemicellulose were quantified by high performance liquid chromatography (HPLC, WATERS), equipped with an Aminex chromatographic column (HPX‐87H, Bio‐Rad, 50 °C), a variable wavelength detector (UV) and a refractive index detector (RID, RI‐H201, Shodex). Dilute sulfuric acid aqueous solution (5 mmol L^−1^) was used as the mobile phase with a flow rate of 0.6 mL min^−1^. All products were quantified by external standards. Phenolic compounds derived from lignin were analyzed by gas chromatography (GC) with a flame ionization detector (Fuli9750), and benzil alcohol was employed as the internal standard. The oven was heated from 50 to 250 °C at a speed of 5 °C min^−1^. The temperature of both detector and sample inlet were kept at 250 °C.

The 2D HSQC NMR spectrum of the liquid fraction was qualitatively determined on spectrometer (ADVANCE 600 MHz, BRUKER). After solvent removal by lyophilization, the samples were completely dissolved in deuterated dimethylsulfoxide (DMSO‐d_6_) for analysis. All chemical displacements were DMSO peaks (δ_C_ 39.5 ppm and δ_H_ 2.5 ppm).

The molecular‐weight distribution of liquid products was analyzed using gel permeation chromatography (GPC, Acquity APC, WATERS) with a TSK gel Super HM−H (6.0 mm×15 cm×2) column and a RI detector. THF was used as the elute at a flow rate of 0.5 mL min^−1^, and the temperature of chromatographic column and differential detector were maintained at 40 °C. Monodisperse polystyrene was used as the standard.

Gas products were collected in aluminum foil bag, in which the air was excluded, and then analyzed by GC (gas chromatography, Folli 9790, Zhejiang Folli Analysis Instrument Co., Ltd.) with TCD as the detector. The temperature of column bin and detector was 120 °C and 160 °C, respectively.

## Conflict of interest

The authors declare no conflict of interest.

1

## Data Availability

The data that support the findings of this study are available from the corresponding author upon reasonable request.

## References

[1] R. Agrawal , A. Verma , R. R. Singhania , S. Varjani , C. D. Dong , A. K. Patel , Bioresour. Technol. 2021, 332, 125042. 33813178 10.1016/j.biortech.2021.125042

[2] M. N. E. H. Belguendouz , J. Gancedo , P. Rapado , D. Ursueguía , Y. Patiño , L. Faba , A. Bahmani , E. Díaz , S. Ordóñez , Chem. Eng. J. 2021, 414, 128902.

[3] X. C. Chen , L. Huang , T. H. A. Chang , B. L. Ong , S. L. Ong , J. Hu , Engineering 2019, 5, 841–848.

[4] S. Xu , R. Wang , T. Gasser , P. Ciais , J. Penuelas , Y. Balkanski , O. Boucher , I. A. Janssens , J. Sardans , J. H. Clark , J. Cao , X. Xing , J. Chen , L. Wang , X. Tang , R. Zhang , Nature 2022, 609, 299–306. 36071193 10.1038/s41586-022-05055-8

[5] J. Liang , B. Li , L. Wen , R. Li , X. Li , Engineering 2021, 7, 203–211.

[6] J. Zhao , F. Yao , C. Hu , Bioresour. Technol. 2022, 358, 127428. 35660654 10.1016/j.biortech.2022.127428

[7] S. Dutta , J. Kim , Y. Ide , J. Ho Kim , M. S. A. Hossain , Y. Bando , Y. Yamauchi , K. C. W. Wu , Mater. Horiz. 2017, 4, 522–545.

[8] J. Xu , L. Dai , Y. Gui , L. Yuan , C. Zhang , Y. Lei , Bioresour. Technol. 2020, 303, 122888. 32028215 10.1016/j.biortech.2020.122888

[9] P. Manzanares , Acta Innovations 2020, 37, 47–56.

[10] W. Liu , C. Liu , P. Gogoi , Y. Deng , Engineering 2020, 6, 1351–1363.

[11] T. Hosoya , H. Kawamoto , S. Saka , J. Anal. Appl. Pyrolysis 2007, 80, 118–125.

[12] W. Yuan , Z. Gong , G. Wang , W. Zhou , Y. Liu , X. Wang , M. Zhao , Bioresour. Technol. 2018, 265, 464–470. 29935456 10.1016/j.biortech.2018.06.038

[13] Y. Luo , Z. Li , X. Li , X. Liu , J. Fan , J. H. Clark , C. Hu , Catal. Today 2019, 319, 14–24.

[14] H. V. Scheller , P. Ulvskov , Annu. Rev. Plant Biol. 2010, 61, 263–289. 20192742 10.1146/annurev-arplant-042809-112315

[15] W. Qi , C. Hu , G. Li , L. Guo , Y. Yang , J. Luo , Y. Du , Green Chem. 2006, 8, 183–190.

[16] S. Bertella , J. S. Luterbacher , Green Chem. 2021, 23, 3459–3467.

[17] A. Corti , E. Torrens , D. Montané , Biomass Conv. Bioref. 2021, https://doi.org/10.1007/s13399-020-01261-4.

[18] S. V. D. Bosch , W. Schutyser , R. Vanholme , T. Driessen , S.-F. Koelewijn , T. Renders , B. D. Meester , W. J. J. Huijgen , W. Dehaen , M. Courtin , B. Lagrain , W. Boerjan , B. F. Sels , Energy Environ. Sci. 2015, 8, 1748–1763.

[19] P. Ferrini , R. Rinaldi , Angew. Chem. Int. Ed. 2014, 126, 8778–8783.

[20] S. Dutta , Carbohydr. Res. 2020, 497, 108140. 32971384 10.1016/j.carres.2020.108140

[21] J. B. G. Filho , R. D. F. Rios , C. G. O. Bruziquesi , D. C. Ferreira , H. F. V. Victória , K. Krambrock , M. C. Pereira , L. C. A. Oliveira , Appl. Catal. B 2021, 285, 119814.

[22] P. Sudarsanam , E. Peeters , E. V. Makshina , V. I. Parvulescu , B. F. Sels , Chem. Soc. Rev. 2019, 48, 2366–2421. 30785143 10.1039/c8cs00452h

[23] R. Rinaldi , Angew. Chem. Int. Ed. 2014, 53, 8559–8560; 10.1002/anie.201404464 25044222

[24] A. R. Morais , A. M. da Costa Lopes , R. Bogel-Łukasik , Chem. Rev. 2015, 115, 3–27. 25411759 10.1021/cr500330z

[25] P. Moniz , H. Pereira , T. Quilhó , F. Carvalheiro , Ind. Crops Prod. 2013, 50, 145–153.

[26] P. M. Grande , J. Viell , N. Theyssen , W. Marquardt , P. D. D. María , W. Leitner , Green Chem. 2015, 17, 3533–3539.

[27] M. A. Mellmer , C. Sener , J. M. Gallo , J. S. Luterbacher , D. M. Alonso , J. A. Dumesic , Angew. Chem. Int. Ed. 2014, 126, 12066–12069. 10.1002/anie.201408359 25214063

[28] T. He , Z. Jiang , P. Wu , J. Yi , J. Li , C. Hu , Sci. Rep. 2016, 6, 38623. 27917955 10.1038/srep38623 PMC5137029

[29] X. Liu , Z. Jiang , S. Feng , H. Zhang , J. Li , C. Hu , Fuel 2019, 244, 247–257.

[30] Z. Jiang , J. Yi , J. Li , T. He , C. Hu , ChemSusChem 2015, 8,1901–1907. 25916895 10.1002/cssc.201500158

[31] A. S. Amarasekara , F. Deng , Biomass Bioenergy 2019, 131, 105421.

[32] F. Yao , F. Shen , X. Wan , C. Hu , Renewable Sustainable Energy Rev. 2020, 132, 110107.

[33] D. M. Alonso , S. G. Wettstein , M. A. Mellmer , E. I. Gurbuz , J. A. Dumesic , Energy Environ. Sci. 2013, 6, 76–80.

[34] J. Zhang , Y. S. Choi , C. G. Yoo , T. H. Kim , R. C. Brown , B. H. Shanks , ACS Sustainable Chem. Eng. 2015, 3, 293–301.

[35] H. Zhang , H. Liu , J. Li , Z. Jiang , C. Hu , ChemSusChem 2018, 11, 1494–1504. 29542869 10.1002/cssc.201800309

[36] Y. Zhou , L. Li , R. Zhang , C. Hu , Faraday Discuss. 2017, 202, 197–212. 28660966 10.1039/c7fd00065k

[37] H. Seddiqi , E. Oliaei , H. Honarkar , J. Jin , L. C. Geonzon , R. G. Bacabac , J. Klein-Nulend , Cellulose 2021, 28, 1893–1931.

[38] H. Wang , G. Gurau , R. D. Rogers , Chem. Soc. Rev. 2012, 41, 1519–1537. 22266483 10.1039/c2cs15311d

[39] S. Yuan , M. V. Tyufekchiev , M. T. Timko , K. Schmidt-Rohr , Cellulose 2022, 29, 2131–2144.

[40] M. Matulova , R. Nouaille , P. Capek , M. Pean , E. Forano , A. M. Delort , Appl. Environ. Microbiol. 2005, 71, 1247–1253. 15746325 10.1128/AEM.71.3.1247-1253.2005 PMC1065164

[41] E. Locci , S. Laconi , R. Pompei , P. Scano , A. Lai , F. C. Marincola , Bioresour. Technol. 2008, 99, 4279–4284. 17920878 10.1016/j.biortech.2007.08.048

[42] P. Sannigrahi , A. J. Ragauskas , S. J. Miller , BioEnergy Res. 2008, 1, 205–214.

[43] N. Brosse , P. Sannigrahi , A. Ragauskas , Ind. Eng. Chem. Res. 2009, 48, 8328–8334.

[44] A. J. Kunov-Kruse , A. Riisager , S. Saravanamurugan , R. W. Berg , S. B. Kristensen , R. Fehrmann , Green Chem. 2013, 15, 2843–2848.

[45] Y. Yang , C. Hu , M. M. Abu-Omar , Green Chem. 2012, 14, 509–513.

[46] A. Tejado , C. Pena , J. Labidi , J. M. Echeverria , I. Mondragon , Bioresour. Technol. 2007, 98, 1655–1663. 16843657 10.1016/j.biortech.2006.05.042

[47] S. S. Y. Tan , D. R. MacFarlane , J. Upfal , L. A. Edye , W. O. S. Doherty , A. F. Patti , J. M. Pringle , J. L. Scott , Green Chem. 2009, 11, 339–345.

[48] K. Yan , G. Wu , T. Lafleur , C. Jarvis , Renewable Sustainable Energy Rev. 2014, 38, 663–676.

[49] Y. Yang , C. Hu , ChemSusChem 2012, 5, 405–410. 22315196 10.1002/cssc.201100688

[50] Y. Yang , C. Hu , M. M. Abu-Omar , Green Chem. 2012, 14, 509–513.

[51] E. M. Anderson , R. Katahira , M. Reed , M. G. Resch , E. M. Karp , G. T. Beckham , Y. Román-Leshkov , ACS Sustainable Chem. Eng. 2016, 4, 6940–6950.

[52] Z. Xue , X. Zhao , R. C. Sun , T. Mu , ACS Sustainable Chem. Eng. 2016, 4, 3864–3870.

[53] Y. Luo , Z. Li , Y. Zuo , Z. Su , C. Hu , J. Agric. Food Chem. 2018, 66, 6094–6103. 29799753 10.1021/acs.jafc.8b01563

[54] Q. Fang , Z. Jiang , K. Guo , X. Liu , Z. Li , G. Li , C. Hu , Appl. Catal. B 2020, 263, 118325.

[55] Z. Chen , W. A. Jacoby , C. Wan , Bioresour. Technol. 2019, 279, 281–286. 30738354 10.1016/j.biortech.2019.01.126

[56] J. Wen , S. Sun , B. Xue , R. Sun , Materials 2013, 6, 359–391. 28809313 10.3390/ma6010359 PMC5452107

[57] A. U. Buranov , G. Mazza , Ind. Crops Prod. 2008, 28, 237–259.

[58] H. Kim , J. Ralph , RSC Adv. 2014, 4, 7549–7560.

[59] Y. Sun , J. Xu , F. Xu , R. Sun , Ind. Crops Prod. 2013, 47, 277–285.

[60] Q. Zhang , G. Wan , M. Li , H. Jiang , S. Wang , D. Min , Int. J. Biol. Macromol. 2020, 162, 236–245. 32535209 10.1016/j.ijbiomac.2020.06.084

[61] R. Li , Y. H. Roos , S. Miao , J. Food Sci. 2017, 82, 2105–2112. 28858389 10.1111/1750-3841.13831

[62] D. Yang , S. Gao , H. Yang , Food Hydrocolloids 2020, 99, 105317.

[63] S. Zinatloo-Ajabshir , M. Baladi , M. Salavati-Niasari , Ultrason. Sonochem. 2021, 72, 105420. 33385636 10.1016/j.ultsonch.2020.105420 PMC7803816

